# The C3-H Bond Functionalization of Quinoxalin-2(1*H*)-Ones With Hypervalent Iodine(III) Reagents

**DOI:** 10.3389/fchem.2020.00582

**Published:** 2020-08-03

**Authors:** Yushi Tan, Jiabo Wang, Hong-Yu Zhang, Yuecheng Zhang, Jiquan Zhao

**Affiliations:** Tianjin Key Laboratory of Chemical Process Safety, Hebei Provincial Key Laboratory of Green Chemical Technology & High Efficient Energy Saving, School of Chemical Engineering and Technology, Hebei University of Technology, Tianjin, China

**Keywords:** hypervalent Iodine(III) reagent, quinoxalin-2(1*H*)-one, C-H functionalization, arylation, trifluoromethylation, alkylation, alkoxylation

## Abstract

The modification of quinoxalin-2(1*H*)-ones *via* direct C-H bond functionalization has begun to receive widespread attention, due to quinoxalin-2(1*H*)-one derivatives' various biological activities and pharmaceutical properties. This mini review concentrates on the accomplishments of arylation, trifluoromethylation, alkylation, and alkoxylation of quinoxalin-2(1*H*)-ones with hypervalent iodine(III) reagents as reaction partners or oxidants. The reaction conditions and mechanisms are compared and discussed in detail.

## Introduction

In recent years, direct C-H functionalization has become one of the most popular topics due to its advantages of having a high bonding efficiency and good atomic economy, and some remarkable achievements having been accomplished in this field (Ackermann et al., 2009; Yang et al., 2017; Yi et al., 2017). Among these significant works, hypervalent iodine reagents have been widely used as ideal and highly efficient oxidants or reaction partners (Kita et al., 1994; Nasrallah et al., 2019) due to their superior bench stability, high reactivity, low toxicity, environmental friendliness, ease of operation, and ready availability (Dohi et al., 2009; Sun and Shi, 2014). For instance, diverse iodine(III) reagents were invented to introduce fluorinated group into organic molecules (Yang et al., 2013; Matsuzaki et al., 2014; Suzuki et al., 2014; Das and Shibata, 2017; Das et al., 2017; Wang et al., 2017). Because of the large size of iodine atoms, a linear three-center, four-electron (3c-4e) bond (L–I–L) which uses a non-hybridized 5p orbital of iodine atom is formed. This 3c-4e bond, termed a “hypervalent bond,” is highly polarized, longer, and weaker than normal covalent bonds, so the hypervalent iodine compounds have high electrophilic reactivity (Zhdankin and Stang, 2008). The distinctive reactivities of hypervalent iodine compounds are similar to those of heavy metals such as lead^IV^, mercury^II^, cadmium^IV^, and thallium^III^. However, compared with heavy metals, iodine is greener and cheaper (Yoshimura and Zhdankin, 2016), and the annual production of iodine reagents is 30,000 tons (Yusubov and Zhdankin, 2015). It is promising that hypervalent iodine compounds can be an environmentally sustainable alternative to heavy metals. The different hypervalent iodine reagents vary in properties. For instances, iodosobenzene and its derivatives have strong oxidability and can replace many toxic oxidants in various oxidation reactions; iodonium salts have no significant oxidative capacity but can react in various ways due to the special leaving ability of the -IAr fragment. Iodonium ylides and imides are excellent carbene and nitrene precursors, respectively, while heterocyclic iodanes have a higher stability than their acyclic analogs, which makes it possible to separate them and makes them a good alternative to several unstable iodine derivatives (Zhdankin and Stang, 2002; Stang, 2003; Wirth, 2005; Küpper et al., 2011).

**Figure 1:**
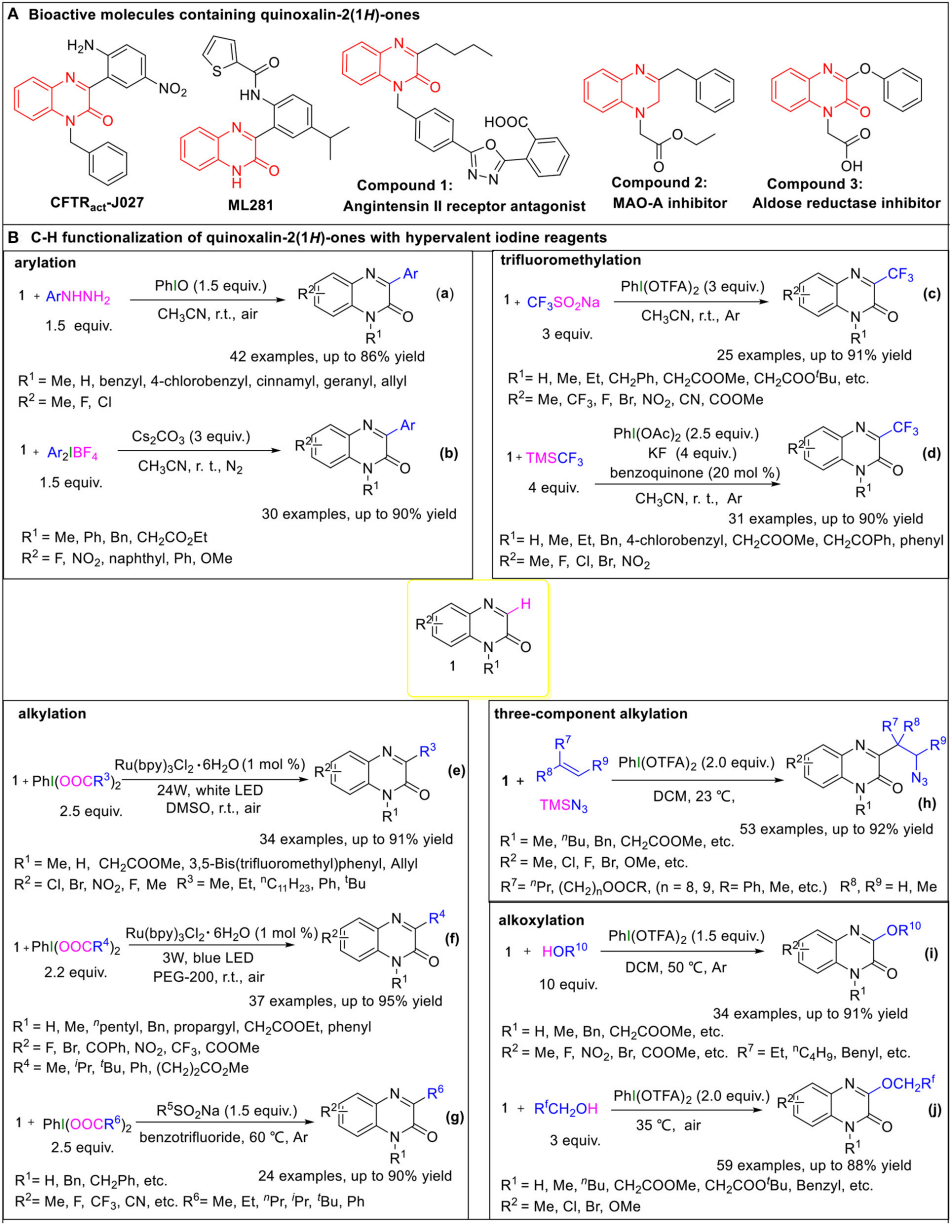
**(A)** Bioactive molecules containing quinoxalin-2(*1*H)-ones. **(B)** C-H functionalization of quinoxalin-2(*1*H)-ones with hypervalent iodine reagents.

On the other hand, quinoxalin-2(1*H*)-one is a privileged structural motif found in various natural active products and drug molecules (Liu et al., 2011; Galal et al., 2014; Pereira et al., 2015). 3-substituted quinoxalinone derivatives specifically have attracted much attention because of their distinctive biological and pharmacological activities. For instance, CFTR_act_-J027 is a safe and efficient CFTR (cystic fibrosis transmembrane conductance regulator) activator which increases intestinal fluid secretion (Cil et al., 2016), and ML281 is a nanomolar STK33 inhibitor that selectively kills KRAS cancers (Weïwer et al., 2012). Some bioactive molecules containing quinoxalin-2(1*H*)-one skeleton, such as **Compounds 1**-**3**, also show potential applications in medicinal chemistry fields (Meyer et al., 2006; Khattab et al., 2015; Qin et al., 2015) (Figure 1A). Because of their synthetic usefulness and potential biological importance, the introduction of functional groups into the C3-position of the quinoxalin-2(1*H*)-ones has already become a research hotspot, and various protocols for the direct C3-H functionalization of quinoxalin-2(1*H*)-ones have been reported (Ebersol et al., 2019; Gu et al., 2019; Hong et al., 2019; Li et al., 2019; Peng et al., 2019; Rostoll-Berenguer et al., 2019; Teng et al., 2019; Wang et al., 2019a, 2020; Xie et al., 2019b; Zhao et al., 2019; Zheng and Studer, 2019; Tian et al., 2020; Yuan et al., 2020). In particular, the C3-H functionalization of quinoxalin-2(1*H*)-ones involving hypervalent iodine reagents has drawn wide attention for the aforementioned advantages of hypervalent iodine reagents, mainly including arylation (Paul et al., 2017; Yin and Zhang, 2017), trifluoromethylation (Wang et al., 2018; Xue et al., 2019a), alkylation (Wang et al., 2019b; Xie et al., 2019a; Xue et al., 2019b; Shen et al., 2020), and alkoxylation (Xu et al., 2019; Yang et al., 2019) of quinoxalin-2(1*H*)-ones, which provide convenient and environmentally friendly means for the synthesis of 3-substituted quinoxalinone derivatives. In this mini review, we will focus on the progress being made in the direct C3-H functionalization of quinoxalin-2(1*H*)-ones involving the hypervalent iodine reagents and discuss their mechanisms, in order to inspire more applications of hypervalent iodine reagents in related reactions.

## C–H Arylation of Quinoxalin-2(1*H*)-Ones Involving Hypervalent Iodine Reagents

Due to the distinctive pharmaceutical and electrical activities of 3-arylquinoxalin-2(1*H*)-ones, various methods for the direct C3-H arylation of quinoxalin-2(1*H*)-ones have been reported, involving two ones taking advantage of hypervalent iodine reagents.

**Figure 2:**
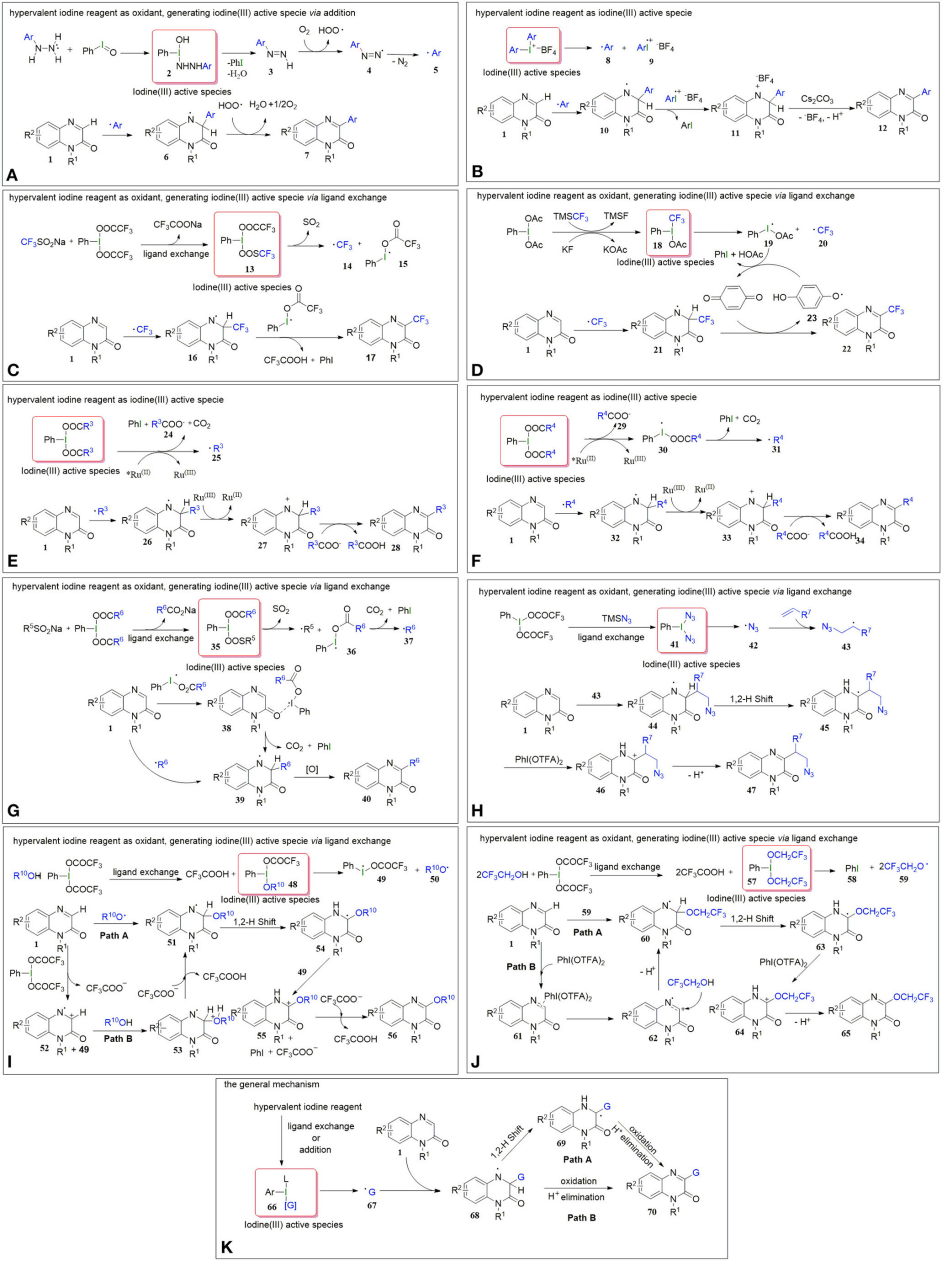
**(A)** The possible mechanism of C-H arylation (a) of Quinoxalin-2(1*H*)-ones. **(B)** The possible mechanism of C-H arylation (b) of Quinoxalin-2(1*H*)-ones. **(C)** The possible mechanism of C-H trifluoromethylationn (c) of Quinoxalin-2(1*H*)-one. **(D)** The possible mechanism of C-H trifluoromethylationn. (d) of Quinoxalin-2(1*H*)-ones. **(E)** The possible mechanism of C-H alkylation (e) of Quinoxalin-2(1*H*)-ones. **(F)** The possible mechanism of C-H alkylation (f) of Quinoxalin-2(1*H*)-ones. **(G)** The possible mechanism of C-H alkylation (g) of Quinoxalin-2(1*H*)-ones. **(H)** The possible mechanism of C-H alkylation (h) of Quinoxalin-2(1*H*)-ones. **(I)** The possible mechanism of C-H alkoxylation (i) of Quinoxalin-2(1*H*)-ones. **(J)** The possible mechanism of C-H alkoxylation (j) of Quinoxalin-2(1*H*)-ones. **(K)** A general mechanism for these reactions.

In March 2017, Paul et al. reported on iodosobenzene-promoted oxidative C3-arylation of quinoxalin-2(1*H*)-ones with arylhydrazines, which is a widely used aryl radical source (Ravi et al., 2015; Rossi et al., 2015) (Figure 1B, Equation a) (Paul et al., 2017). The transformation afforded a variety of 3-arylquinoxalin-2(*H*)-one derivatives in moderate to good yields with a broad substrate scope, and the reaction conditions were mild, using only iodosobenzene as the oxidant. This protocol developed a new system for the synthesis of 3-arylquinoxalin-2(1*H*)-one derivatives. The aryl-TEMPO adduct was detected in the presence of the radical trapping reagent (2,2,6,6-tetramethylpiperidin-1-yl)oxyl (TEMPO) under the standard conditions, indicating that an aryl radical was involved in the reaction. Based on the experimental results, a plausible mechanism was proposed by the authors (Figure 2A). First, arylhydrazine adds to PhIO to form the iodine(III) active species **2**, which then eliminates the H_2_O and PhI to give aryldiazene **3**. Subsequently, the intermediate **3** is oxidized by oxygen to afford the diazenyl radical **4**. The radical **4** releases molecular nitrogen to generate aryl radical **5**, which reacts with quinoxalin-2(*H*)-one **1** to form the intermediate **6**. Eventually, the intermediate **6** is oxidized by the hydroperoxyl radicals generated in the process of the formation of **4** to provide the final product **7**.

In the same year, Zhang group reported a method for C3-H arylation of quinoxalin-2(1*H*)-ones to give 3-arylquinoxalin-2(1*H*)-ones with diaryliodonium salts (Figure 1B, Equation b) (Yin and Zhang, 2017). Diaryliodonium salts are generally used in organic synthesis as aryl radical sources due to their easy availability (Yamaoka et al., 2013; Wang D. et al., 2014). This protocol could tolerate a series of readily available diaryliodonium salts and quinoxalin-2(1*H*)-ones, indicating its broad substrate scope. A possible mechanism was proposed for the coupling reaction based on the results of radical trapping with TEMPO (Figure 2B). Initially, aryl radical **8** is generated *via* the decomposition of diaryliodonium salt, and then immediately adds to quinoxalin-2(1*H*)-one to form the nitrogen radical **10**. The nitrogen radical **10** is then oxidized by high-valence iodonium salt **9** to produce the intermediate **11**, which eliminates the H^+^ and ${\text{BF}}_{4}^{-}$ in the presence of Cs_2_CO_3_ to give the final product **12**.

## C–H Trifluoromethylation of Quinoxalin-2(1*H*)-Ones Involving Hypervalent Iodine Reagents

Because of the special biological and drug activities of 3-trifluoromethylquinoxalin-2(1*H*)-one derivatives (Patel et al., 2000; Carta et al., 2006), it is necessary to explore efficient, simple, and mild methods to synthesize the 3-trifluoromethylquinoxalin-2(*H*)-ones. In 2018, we employed sodium trifluoromethanesulfinate (Langlois reagent) as a trifluoromethyl source and successfully realized the C3-H trifluoromethylation of quinoxalin-2(*H*)-ones under mild and transition metal-free conditions (Figure 1B, Equation c) (Wang et al., 2018). Due to the low-cost of sodium trifluoromethanesulfinate and easy availability of hypervalent iodine reagents, this protocol offers simple, efficient, and cheap access to 3-trifluoromethylquinoxalin-2(*H*)-ones. When the reaction was performed in the presence of the radical trapping reagent 1,1-diphenylethylene (DPE), the target product (3-trifluoromethylquinoxalin-2(*H*)-one) was isolated only in a 46% yield, and a DPE-CF_3_ coupled byproduct (3,3,3-trifluoroprop-1-ene-1,1-diyl)dibenzene was observed, which suggested that the reaction followed a radical mechanism (Figure 2C). First, [bis(trifluoroacetoxy)iodo]benzene (PhI(OTFA)_2_) undergoes anion exchange with sodium trifluoromethanesulfinate to afford iodine(III) active species **13**, which then releases sulfur dioxide to generate trifluoromethyl radical **14** and I-radical **15**. Thereafter, trifluoromethyl radical **14** reacts with quinoxalin-2(1*H*)-ones to afford the intermediate **16**, which eventually goes through oxidization and H-elimination to form the final product **17**.

Soon afterwards, in March 2019, Xue et al. reported a method to synthesize 3-trifluoromethylquinoxalin-2(1*H*)-ones with (trifluoromethyl)trimethylsilane (Ruppert-Prakash reagent) as a trifluoromethyl source under transition metal-free conditions (Figure 1B, Equation d) (Xue et al., 2019a). This protocol has the advantages of using cheaper (diacetoxyiodo)benzene(PhI(OAc)_2_)compared with PhI(OTFA)_2_ as an oxidant and stable (trifluoromethyl)trimethylsilane as the trifluoromethyl source. When TEMPO was added into the model reaction as a radical scavenger, the desired transformation was completely suppressed, while the TEMPO-CF_3_ adduct was obtained in a 70% yield. Based on this experiment's results, the following possible mechanism was proposed (Figure 2D). Initially, the PhI(OAc)_2_ reacts with KF and TMSCF_3_ to generate the iodine(III) active species **18**, which then decomposes to I-radical **19** and trifluoromethyl radical **20**. Subsequently, trifluoromethyl radical **20** attacks quinoxalin-2(1*H*)-one to form intermediate **21**, which then oxidizes by 1,4-benzoquinone to form the final product **22**, with the 1,4-benzoquinone converting to phenoxy radical **23**. Finally, the phenoxy radical **23** recovers to 1,4-benzoquinone *via* oxidation of the I-radical **19**.

## C–H Alkylation of Quinoxalin-2(1*H*)-Ones Involving Hypervalent Iodine Reagents

As we know, phenyliodine(III) dicarboxylates have been regarded as effective reagents for the introduction of alkyl groups into organic molecules through a radical decarboxylation procedure (Togo and Katohgi, 2001; Lu et al., 2018). Besides, phenyliodine(III) dicarboxylate reagents could be easily prepared and stored (Stang et al., 1988; Mocci et al., 2007). So, several strategies for the synthesis of 3-alkylquinoxalin-2(1*H*)-ones with phenyliodine(III) dicarboxylates as the alkyl sources have been developed.

Very recently, Xue et al. developed a method to synthesize 3-alkylquinoxalin-2(1*H*)-ones with phenyliodine(III) dicarboxylates under visible-light conditions (Figure 1B, Equation e) (Xue et al., 2019b). The use of cheap and readily available phenyliodine(III) dicarboxylates as the alkylation reagents and mild reaction conditions make this protocol convenient and efficient in the synthesis of 3-alkylquinoxalin-2(1*H*)-ones. Two control experiments were carried out to probe into the mechanism. One was to use DMSO-*d*6 instead of DMSO as solvent. In this case the product was 3-methylquinoxalin-2(1*H*)-one but not the deuterated methyl substituted quinoxalin-2(1*H*)-one, which illustrated that the methyl group came from PhI(OAc)_2_ rather than solvent DMSO. The other one was to run the reaction in the presence of TEMPO under the standard conditions. The TEMPO-CH_3_ adduct was detected, indicating that the reaction was a radical process. Based on these control experiments, a plausible mechanism for this alkylation was proposed, as shown in Figure 2E. First, the catalyst Ru(II) is irradiated by visible light to produce the excited state ^*^Ru(II), which promotes the decarboxylation of PhI(OOCR^3^)_2_ to generate alkyl radical **25**. Subsequently, the alkyl radical **25** regio-selectively attacks the quinoxalin-2(1*H*)-ones to provide intermediate **26**, which oxidizes by Ru(III) to transform into cation **27**, regenerating catalyst Ru(II). Eventually, the cation **27** undergoes dehydrogenation with the assistance of carboxylate anion **24** to furnish the final product **28**.

In the same year, He and coworkers also reported a method for the synthesis of 3-alkylquinoxalin-2(1*H*)-ones utilizing phenyliodine(III) dicarboxylates as the alkyl sources under visible-light conditions (Figure 1B, Equation f) (Xie et al., 2019a). And, more remarkable, this protocol uses eco-friendly PEG-200 as a reaction solvent. Based on the radical trapping results with dibutylhydroxytoluene (BHT) or TEMPO, a probable mechanism similar to the former was proposed (Figure 2F).

Alternatively, we accomplished the synthesis of 3-alkylquinoxalin-2(1*H*)-ones with phenyliodine(III) dicarboxylates as alkyl agents mediated by sodium alkylsulfinates under mild conditions (Figure 1B, Equation g) (Wang et al., 2019b). This protocol does not need any expensive metal catalysts, endowing it with an efficient process for the synthesis of 3-alkylquinoxalin-2(1*H*)-ones without metallic residues. Based on radical trapping experimental results with TEMPO and previous studies, a plausible mechanism was proposed, as given in Figure 2G. Initially, the anion exchange of PhI(O_2_CR^6^)_2_ with R^5^SO_2_Na generates the iodine(III) active species **35**, which then turns into radical R^5^ and I-radical **36**, excluding SO_2_ at the same time. Then I-radical **36** decomposes into alkyl radical **37** and PhI, along with the release of CO_2_. Next, in the presence of I-radical **36**, quinoxalin-2(1*H*)-one transforms into intermediate **38**, which undergoes decarboxylation to generate intermediate **39**. Meanwhile, the attack of alkyl radical **37** on the quinoxalin-2(1*H*)-one can also produce intermediate **39**, which is another path to producing intermediate **39**. Finally, intermediate **39** transforms to final product **40** *via* oxidization and dehydrogenation.

In short, the above three protocols for the alkylation of quinoxalin-2(1*H*)-ones showed superiority in functional group tolerance under mild reaction conditions. And yet the first two featured the efficient and sustainable visible-light-induced reaction systems, while the third one displayed the character of transition metal-free conditions.

## Three-Component C–H Alkylation of Quinoxalin-2(1*H*)-Ones Involving Hypervalent Iodine Reagents

In 2020, Zhang's group developed a hypervalent iodine(III)-promoted three-component alkylation of quinoxalin-2(1*H*)-ones with unactivated alkenes and TMSN_3_ (Figure 1B, Equation h) (Shen et al., 2020). This method provides a step-economical solution for the introduction of β-azido alkyl groups into the quinoxalin-2(1*H*)-ones to rapidly synthesize bioactive organoazides. The various substituted quinoxalin-2(1*H*)-ones bearing electron-rich or electron-deficient groups, and olefins with functional groups including ester or hydroxyl substituents, were well-compatible in this transformation. A radical mechanism is proposed in Figure 2H. A double ligand exchange between PhI(OTFA)_2_ and TMSN_3_ furnishes iodine(III) active species, which provides an azide radical (**42**) *via* the homolytic cleavage of I-N bond (Matcha et al., 2013). Then, **42** chemoselectively attacks olefins to generate alkyl radical intermediate **43**, which is trapped by substrate **1** to afford nitrogen radical intermediate **44**. Afterwards, **44** undergoes a 1,2-H shift process to afford carbon radical **45**. Finally, the target product **47** was obtained *via* the sequential procedures of oxidation and deprotonation from **45**.

## C–H Alkoxylation of Quinoxalin-2(1*H*)-Ones Involving Hypervalent Iodine Reagents

Alkoxy groups are widely present in various natural active products and drug molecules (Han et al., 2018; Zhang et al., 2019), but the methods for the introduction of alkoxy groups in quinoxalin-2(1*H*)-ones have been rarely reported. In 2019, we disclosed a method to synthesize 3-alkoxyquinoxalin-2(1*H*)-ones with alcohols and PhI(OTFA)_2_ under mild conditions (Figure 1B, Equation i) (Yang et al., 2019). This protocol provides easy access to 3-alkoxyquinoxalin-2(1*H*)-ones by using readily available and low cost alcohols as the alkoxy sources, and cheap PhI(OTFA)_2_ as the oxidant, and shows good functional-group tolerance. Similarly, either BHT or TEMPO was employed as the radical trapping agent to explore information on the reaction mechanism. The results indicated that a radical mechanism could be involved in this alkoxylation process (Figure 2I). First, the ligand exchange of PhI(OTFA)_2_ with alcohol takes place to afford the iodine(III) active species **48**, which then decomposes into I-radical **49** and alkoxy radical **50**. Subsequently, alkoxy radical **50** adds to quinoxalin-2(1*H*)-ones to generate the nitrogen radical **51**. There is also another way to form the intermediate **51**, namely, quinoxalin-2(1*H*)-one reacts with PhI(OTFA)_2_ to generate radical cation **52**, and radical cation **52** is captured by nucleophilic alcohols to form oxonium intermediate **53** which undergoes deprotonation to afford the intermediate **51** with the assistance of trifluoroacetate. The nitrogen radical **51** converts to carbon radical **54** *via* 1,2-H shift process, which goes through oxidation and deprotonation successively to transform into final product **56**.

In addition, fluorine-containing units have been found in various drugs and nature products and are widely used in the pharmaceutical industry and in material science (Liang et al., 2013; Wang J. et al., 2014). Fluoroalkoxyl aryl ethers have also become a research hotspot because of their special pharmaceutical and biological activities. In June 2019, Zhang group reported a method to synthesize 3-fluoroalkoxylquinoxalin-2(1*H*)-ones with fluoroalkyl-alcohols and PhI(OTFA)_2_ under catalyst-free and solvent-free conditions (Figure 1B, Equation j) (Xu et al., 2019). The use of commercially available fluoroalkyl alcohols as the fluoroalkoxyl sources, and convenient PhI(OTFA)_2_ as the oxidant in the absence of a catalyst and solvent, endows this novel strategy with environmental friendliness and efficiency for the direct C3-H fluoroalkoxylation of quinoxalin-2(1*H*)-ones. The observation of TEMPO-OCH_2_CF_3_ or DPE-OCH_2_CF_3_ adducts in the radical trapping experiments revealed that a radical pathway may be involved in the reaction. The probable mechanism of the fluoroalkoxylation is similar to the aforementioned alkoxylation of quinoxalin-2(1*H*)-ones (Figure 2J).

## Conclusion

In this mini review, we summarized recent efforts on direct C3-H functionalization of quinoxalin-2(1*H*)-ones with the commercially available and environmentally benign hypervalent iodine reagents, mainly including arylation, trifluoromethylation, alkylation, and alkoxylation. The accomplishments have provided us with simple, mild, efficient, and eco-friendly methods for the synthesis of various C3-substituted quinoxalin-2(1*H*)-ones. Herein, the hypervalent iodine reagents play two different roles, reaction partners (**equations b**, **e**, **f**, and **g**), and oxidants (**equations a**, **c**, **d**, **h, Io**, and **j**). A general mechanism for these reactions could be given (Figure 2K). For all the reactions, iodine(III) active species are initial key intermediates. In the case of hypervalent iodine reagents as oxidants, they must initially transform to the iodine(III) active species **66** *via* ligand exchange (**equations c**, **d**, **h**, **i**, and **j**) or addition (**equation a**); in the case of hypervalent iodine reagents as reaction partners, they are the iodine(III) active species **66** themselves (**equations b**, **e**, **f**, and **g**). Once the formation or introduction of iodine(III) active species occurs, they decompose to afford the radical **67** (G = aryl, trifluoromethyl, alkyl, azide and alkoxyl radical), which regio-selectively adds on to the N=C bond of quinoxalin-2(1*H*)-one to provide the carbon radical intermediate **68** (not including the azide radical). In some situations, the intermediate **68** transforms into nitrogen radical intermediate **69** *via* a 1,2-*H* shift process. Finally, the target product **70** is obtained *via* the successive oxidation and H^+^-elimination of the carbon radical intermediate **68** or nitrogen radical intermediate **69**.

There are still some limitations in the reactions involving hypervalent iodine reagents. For instance, superstoichiometric hypervalent iodine reagents or noble transition-metal-catalysts are required in some cases. Also, the application scope of hypervalent iodine reagents is slightly narrow; they have only been successful in the arylation, trifluoromethylation, alkylation, and alkoxylation of quinoxalin-2(1*H*)-ones up till now. It is desirable to develop efficient reaction systems and expand on more reaction models such as the acylation, alkoxycarbonylation, amination, sulfonation, and phosphonation of quinoxalin-2(1*H*)-ones involving hypervalent iodine reagents.

## Author Contributions

YT, JW, and H-YZ collected the related references and prepared the manuscript. YZ and JZ directed the preparation of this manuscript. All authors critically reviewed the text and figures prior to submission.

## Conflict of Interest

The authors declare that the research was conducted in the absence of any commercial or financial relationships that could be construed as a potential conflict of interest.
